# On the Localisation of the Seat of Hæmorrhage in Hæmaturia

**Published:** 1899-04-29

**Authors:** J. Lacy Firth

**Affiliations:** Assistant Surgeon to the Bristol General Hospital


### Hospital Clinics and Medical Progress.

### ON THE LOCALISATION OF THE SEAT OF HÆMORRHAGE IN HÆMATURIA.

By J. Lacy Firth, M.D., M.S., F.R.C.S., Assistant Surgeon to the Bristol General Hospital.

In attempting to diagnose the nature of a lesion which causes hæmaturia, it is advisable in the first place to endeavour to localise the source of the hæmorrhage, to discover whether it lie in the kidney, ureters, bladder, prostate, or urethra. Rules have been formulated for the purpose of helping the practitioner to correctly determine this point. They are the following :—

Rule I.—*The brighter the tint of the blood in the urine, the nearer is the source of it to the meatus.*

This rule is generally reliable, but in renal carcinoma and sarcoma, and in severe injuries of the kidney, the hæmorrhage may be so free and the blood be so quickly expelled from the bladder that its colour remains bright. In other cases the rule holds good, and the passage of bright blood in urine indicates that the hæmorrhage is of urethral, prostatic, or vesical origin.

The converse of this rule, however, that dark blood in urine is of renal origin must by no means be assumed. Urine tends to change the colour of hæmoglobin to a dark hue—to brown, like porter, or to black. Further, if a large quantity of urine be intimately mixed with a small quantity of blood, the well-known "smoky" tinted urine is produced. Naturally, it takes time for urine to induce the chemical change which darkens the red colour of hæmoglobin. Any cause which increases the time elapsing between the formation of the mixture of the blood and urine and its discharge from the body will tend to increase the degree of this change of colour. It naturally follows that if the mixture is made in the kidney at the time or soon after the urine is secreted, it is likely to be dark in colour when discharged. Smoky urine is, therefore, often of renal origin, *e.g.*, it is common in acute nephritis. But urine of the same appearance is easily produced by the slow oozing of blood into the bladder from any source, *e.g.*, from a vesical growth or ulcer. Again, whatever be the source of blood passed into the bladder, if it be retained there in contact with urine, it will be darkened. The existence of any degree of atony of the bladder, frequent in cases of stricture of the urethra and enlargement of the prostate with retention of residual urine, will therefore prevent the expulsion of bright-coloured blood. Similarly, if blood undergoes clotting in the bladder, it will become darkened if the clotting delays its expulsion.

Therefore, though bright blood in the urine indicates urethral or vesical hæmorrhage, dark blood does not indicate, with any accuracy, the source of the hæmorrhage; it merely indicates that a considerable time has elapsed since the blood and urine were mixed. It is evident from the above considerations that the colour of the urine in hæmaturia is not often a very reliable guide to the source of hæmorrhage. Co-existing symptoms, the microscopical examination of the urine, and other aids described below, will afford more assistance in the localisation of the source of hæmorrhage.

Rule II.—*Blood appearing at or near the end of micturition has a vesical or prostatic origin.*

This is a reliable rule, and the explanation is simple. The blood circulating in the mucous membrane of the bladder leaves by veins which pass through the muscular planes of the bladder wall. When the bladder contracts these veins of exit become compressed, and this tends to produce engorgement of the mucous membrane. If the latter be thinned by ulceration, or if there be the thin-walled vessels of a villous growth present, blood is likely to escape as the bladder contracts. If for any reason there is straining with micturition, then the tendency to the production of this kind of hæmorrhage will be proportionately greater.

Rule III.—*Fluid blood appearing at the beginning of micturition is of prostatic origin.*

The word fluid is introduced into the rule because a clot of *vesical* origin may settle over the opening of the urethra into the bladder and escape with the first rush of urine. If the blood first expelled is a clot which has been moulded in the prostatic urethra, it is likely to have an ovoid leech-like form. If it be a clot which has been moulded in the urethra anterior to the prostate it may be long and bougie-like. In addition it must be remembered that prostatic blood, if profusely discharged, may flow back into the bladder and tinge the whole quantity of urine. Also that a lesion at the neck of the bladder may lead to the expulsion of a clot at the beginning of micturition, followed by a flow of clear urine, and ending with the reappearance of blood caused as described under Rule II.

Rule IV.—*Blood escaping from the meatus independently of micturition is of urethral origin.*

Rule V.—*Long thin clots like earth-worms, or smaller thin clots like red fishing worms, passed in urine, have been moulded in the ureters and indicate that the hæmorrhage has been renal.*

Sometimes the escape of one of these clots is a herald of the immediate recurrence of a hæmaturia which has ceased. In such a case the cessation of hæmorrhage has been due to the plugging of the ureter with clot, and the recurrence is due to the tension of the accumulating blood and urine above the clot finally dislodging it. Hurry Fenwick has recorded a case in which every attack of hæmaturia was preceded by the escape of a worm-like clot. Cystoscopic examination showed the lower end of such a clot protruding from the ureteral orifice. After removing it by suction, scarlet blood was immediately seen to issue from the same orifice.

Rule VI.—*Large, irregular edged, eroded clots are usually derived from a bladder source.*

Clots are seldom found in urine unless hæmorrhage has been free. They should be looked for immediately after micturition, as urine soon dissolves them. Usually, their colour is dark red, but may be bright red or black, or they may be almost completely decolourised. They are often very irregular in size and shape. The bleached clots are especially liable to be mistaken for fragments of growth (Newman). The absence of nuclei in the decolourised discs and other microscopical features will serve to distinguish them. There are important exceptions to the rule that large irregular clots have a vesical origin, though it holds good for a large proportion of cases. In severe traumatic lesions of the kidney, and in renal sarcoma and carcinoma, if hæmorrhage is free, the bladder may fill with large clots. Also blood from a granular kidney, or from a kidney acutely or subacutely inflamed may clot in the bladder, or may clot in the pelvis of the kidney and subsequently pass into the bladder. Newman states that he has seen a clot passed from the bladder which formed almost a complete cast of the renal pelvis and uppermost part of the ureter, and that in another case he found clots in the pelves of both kidneys extending down the ureters and projecting into the bladder. It may be mentioned that the clotting of blood in urine which is not free from septic organisms is a dangerous condition, for there is great danger of the septic decomposition of the clots infecting the kidneys through the ureters. The strictest antiseptic precautions must therefore always be taken if instruments, such as catheters or evacuators, are used to remove clots which the bladder is unable to expel.

It will have been noticed that of the above six rules, the first is based upon the colour of the urine; the second, third, and fourth upon the time in relation to micturition at which the blood appears at the meatus; and the fifth and sixth upon the characters of the clots.

Other aids to the discovery of the source of bleeding in hæmaturia are based upon—(*a*) the mode of subsidence of the urine on standing; (*b*) the admixture with other urinary deposits; (*c*) cystoscopic appearances; (*d*) the character of the urine obtained by the catheterisation of the ureters; (*e*) the relative quantities of albumen and hæmoglobin in the urine.

(*a*) The Mode of Subsidence of the Urine: Von Jaksch has made rather precise statements on this point. He says that if blood is intimately mixed with urine even though the tint is deep, and the red corpuscles do *not* fall to the bottom of the test glass, then the hæmorrhage is renal, pelvic, or ureteral; and further, if the discs have lost their colouring, and are pale yellow discs, the hæmorrhage is renal, and due to acute nephritis or to an exacerbation in the course of chronic nephritis. Since these particular features of the secretion depend upon the reaction and other characters of the urine, the completeness of the admixture with blood, and the length of time that has elapsed since the mixture was made, it seems *a priori* likely that they would not be so special to hæmaturia of renal or pelvic origin as stated. Dr. Newman[1] states that he has seen the urine in cases of hæmaturia vary much from day to day, sometimes having the character just described, though the hæmorrhage subsequently proved not to be of renal origin.

If there be much pus in the urine together with blood, the origin being renal or pelvic, often the pus will subside to the bottom of the glass, carrying with it almost all the blood cells. Excess of mucous points to vesical catarrh, and often then the blood is at the bottom of the glass and the mucous above it, the latter being uniformly stained or merely streaked with red.

(*b*) The Admixture of Other Deposits: The most important of these, in addition to the pus and mucous already mentioned, which are of significance in localising the source of hæmorrhage, is that with fragments of growth recognisable under the microscope. It is said that these fragments are only found in cases of vesical neoplasm, and apparently more frequently in the benign growths than in the malignant.

(*c*) Cystoscopy: This mode of examination of the bladder will definitely locate the source of hæmorrhage if a vesical lesion is seen, or if blood is seen issuing from one or both ureters.

(*d*) Catheterisation of the Ureters: This difficult proceeding is not of great value for the purpose of locating the hæmorrhage, for the necessary manipulations may themselves excite ureteral hæmorrhage. Nor is it necessary, as the cystoscope will usually show if blood is escaping from a ureter.

(*e*) Comparison of the Quantity of Albumen with that of Hæmoglobin: Dr. David Newman has shown the value of this comparison. Urine which contains blood of necessity contains albumen. If the escape of blood is associated with inflammation, then the quantity of albumen present will be greater than can be accounted for by the presence of blood. If the ratio of albumen to hæmoglobin be 1—1·6, it may be concluded that all the albumen is due to the blood; but if the albumen is present in a greater proportion than that, it points to the existence of independent albuminuria, and to a renal inflammation as the cause.

The albumen is estimated in one of the usual ways. To estimate the hæmoglobin use is made of the fact that in solutions of that substance, of a certain degree of dilution, viz., 1 in 22,000 of water, one of the two absorption bands in the spectrum, that near Fraunhofer's line E is lost, if the film is of a certain thickness. A hæmatoscope is used, and the thickness of the film of urine which causes this absorption band near E to be lost is measured. A simple calculation then gives the percentage of hæmoglobin present.

